# Enhanced performance of mixed HWMA-CUSUM charts using auxiliary information

**DOI:** 10.1371/journal.pone.0290727

**Published:** 2023-09-15

**Authors:** Faiza Zubair, Rehan Ahmad Khan Sherwani, Muhammad Abid

**Affiliations:** 1 Higher Education Department, Lahore, Pakistan; 2 University of the Punjab, Lahore, Pakistan; 3 Government College University, Faisalabad, Pakistan; Max Planck Institute for Solid State Research, GERMANY

## Abstract

Quality control (QC) is a systematic approach to ensuring that products and services meet customer requirements. It is an essential part of manufacturing and industry, as it helps to improve product quality, customer satisfaction, and profitability. Quality practitioners generally apply control charts to monitor the industrial process, among many other statistical process control tools, and to detect changes. New developments in control charting schemes for high-quality monitoring are the need of the hour. In this paper, we have enhanced the performance of the mixed homogeneously weighted moving average (HWMA)-cumulative sum (CUSUM) control chart by using the auxiliary information-based (AIB) regression estimator and named it MHC_AIB_. The proposed MHC_AIB_ chart provided an unbiased and more efficient estimator of the process location. The various measures of the run length are used to judge the performance of the proposed MHC_AIB_ and to compare it with existing AIB charts like CUSUM_AIB_, EWMA_AIB_, MEC_AIB_ (mixed AIB EWMA-CUSUM), and HWMA_AIB_. The Run length (RL) based performance comparisons indicate that the MHC_AIB_ chart performs relatively better in monitoring small to moderate shifts over its competitor’s charts. It is shown that the chart’s performance improves with the increase in correlation between the study variable and the auxiliary variable. An illustrative application of the proposed MHC_AIB_ chart is also provided to show its implementation in practical situations.

## 1. Introduction

Statistical process control and monitoring (SPCM) consists of several statistical tools, and control charts are considered the most efficient. The control charts resolve irregular deviations from the required standards in manufacturing and industrial processes. The memory-less and memory types are the two core divisions of the control charts (cf. Montgomery [1]). Shewhart [2] proposed memory-less control charts, which use only current sample information for process monitoring. The memory type charting procedures, for instance, the cumulative sum (CUSUM), the exponentially weighted moving average (EWMA), the progressive mean (PM), and the homogeneously weighted moving average (HWMA) were developed by Page [3], Roberts [4], Abbas et al. [5] and Abbas [6] respectively and the monitoring statistics of these charts grasp earlier sample information along with the recent information.

On control charts, various types of extensions have been introduced in the literature on the SPCM. Combining two control charts also improved the efficiency of the control charts. Lucas [7] and Lucas and Saccucci [8] suggested the combined design structure of the Shewhart-EWMA and Shewhart-CUSUM charts, respectively. Shamma and Shamma [9] proposed a double EWMA chart. Mixed design structures of EWMA-CUSUM (MEC) and CUSUM-EWMA (MCE) charts were suggested by Abbas et al. [10] and Zaman et al. [11] respectively. Motivated by the study of Shamma and Shamma [8], double PM and HWMA charts were suggested by Abbas et al. [12] and Abid et al. [13] respectively. A mixture of PM and EWMA charts was proposed by Abbas et al. [14]. Taking inspiration from Abbas et al. [9] and Abid et al. [15] developed a mixed HWMA-CUSUM (MHC) chart in which statistic of the CUSUM chart runs as the output and MHC chart outperforms against the EWMA, HWMA, and MEC charts.

In sample surveys, the precision of the estimates of the population parameters can be increased by using auxiliary information. The auxiliary variable is a variable known for all units of the population but not a variable under study. The auxiliary information-based (AIB) charts are usually developed using regression and ratio estimators to monitor the process variable effectively. In the SPCM literature, much work has been done related to the AIB charts. Riaz [16]and Riaz [17] proposed a regression estimator-based Shewhart _(AIB)_ chart for monitoring process location and dispersion, respectively. The regression EWMA_AIB_ chart was proposed by Abbas et al. [18] and the EWMA_AIB_ performed well against the usual EWMA chart without the AIB information. Abbas [19] suggested the CUSUMAIB chart performed relatively better than the usual CUSUM chart. Ahmad et al. [20] suggested some AIB charts for the autocorrelated processes. Adegoke et al. [21] designed a regression HWMA_AIB_ chart when the process variable is investigated under normal and non-normal environments and revealed that the HWMA_AIB_ chart is more powerful than the EWMA_AIB_ and CUSUM_AIB_ charts. Sanusi et al. [22] suggested various ratio estimators based on EWMA charts. The regression PM_AIB_ chart was suggested by Abbas et al. [12] under zero-state and steady-state processes. Interested readers can see the work of Ahmad et al. [20], Haq and Khoo [23], Abbasi and Haq [24], Noor-ul-Amin et al. [25], and Hussain et al. [26] on AIB charts. Dirbaz et al. [27] suggested two new AIB-based control charts, AIB-MEWMA and AIB-DMEWMA charts, to detect shifts in model parameters. Arslan et al. [28] designed a sensitive homogeneously weighted moving average chart using two supplementary variables (hereafter, TAHWMA), which is an efficient and unbiased estimator for the process mean if the two supplementary variables correlate with the study variable.

In the SPCM literature, very little work is available on AIB mixed memory control charts. Recently, Anwar et al. [29] designed a regression estimator based MEC_AIB_ and MCE_AIB_ charts for prompt detection of persistent changes, and the MEC_AIB_ chart is more effective than the MCE_AIB_ chart and as well as the EWMA_AIB_ and CUSUM_AIB_ charts. Adegoke et al. [21] revealed that the performance of the regression estimator is relatively better than the ratio estimator. The core focus of this study is to propose an efficient mixed memory chart under the scenario of the regression estimator. So, this study proposes a new regression estimator based MHC chart labeled as MHC_AIB_ for detecting persistent deviations in the process location. The MHC_AIB_ chart is a mixture of the HWMA_AIB_ and the usual CUSUM chart. In the recent age of development, improvements in quality assurance techniques are the need of the hour. In context, we have developed a new chart showing visible improvement in detecting shifts. Even a minute change and deviation in the quality can be a big hurdle in many industrial processes like lifesaving drugs, substrate manufacturing, missile equipment, etc. In some industrial and manufacturing processes, the auxiliary information is also recorded along with the under-study variables for different tasks. This information can be used to improve the control chart design without imparting any additional financial burden to the entrepreneur. We can use this information for the improvement of design. Our proposed chart is shown to have improved results compared to its counterparts and can be used for high-quality monitoring in different industrial applications.

The rest of the article is outlined as follows: the next section offers the structure of the MHC and the MHC_AIB_ charts, along with the RL evaluation of the proposed MHC_AIB_ chart. The performance evaluation and RL comparisons of the MHC_AIB_ chart against the competitor’s charts are delivered in Section 3. A numerical example of the MHC_AIB_ and existing charts are offered in Section 4, Section 5 gives the limitation of the study, and the article ends with a conclusion and recommendations.

## 2. The Mixed HWMA-CUSUM (MHC) and the proposed Mixed HWMA-CUSUM with auxiliary information (MHC_AIB_) control charts

This section includes a description of the construction of the existing Mixed HWMA-CUSUM (MHC) and the proposed Mixed HWMA-CUSUM with additional information MHC_AIB_ charts:

### 2.1. The MHC chart

Let *z*_*ij*_ is the variable of interest follows the normal distribution, i.e., $z_{ij}\sim N\left(\mu_{z},\;\sigma_{z}^{2}\right)$ where *μ*_*z*_ and $\sigma_{z}^{2}$ is the in-control (IC) mean and variance of the process variable, respectively, *i* = 1, 2, 3, … and *j* = 1, 2, 3, …, *n*. Abbas [6] suggested the statistic of the HWMA chart as follows:

$$H_{i}=\theta {\bar{z}}_{i}+\left(1-\theta \right){\bar{\bar{z}}}_{i-1}$$ (1)

Where *θ* is the smoothing parameter (*θ* ∈ (0, 1]), ${\bar{\bar{z}}}_{0}=\mu_{z}$, and ${\bar{\bar{z}}}_{i-1}=\frac{\sum_{m=1}^{i-1}{\overset{-}{Z}}_{m}}{i-1}$. The IC mean and variance for *H*_*i*_ are as follows (cf. Abbas [6]):

$$\left.\begin{array}{c} Mean\left(H_{i}\right)=\mu_{z}\\ Var\left(H_{i}\right)=\frac{\theta^{2}\sigma_{z}^{2}}{n} \end{array}if\;i=1\right)\;\;\text{And}\;\left.\begin{array}{c} Mean\left(H_{i}\right)=\mu_{z}\\ Var\left(H_{i}\right)=\frac{\theta^{2}\sigma_{z}^{2}}{n}+{\left(1-\theta \right)}^{2}\frac{\sigma_{z}^{2}}{n\left(t-1\right)} \end{array}if\;i>1\right)$$

Abid et al. [15] proposed the MHC chart by placing the statistic given in (1) with the CUSUM statistic, and the plotting statistics of the MHC chart are given as:

$$\left.\begin{array}{c} MHC_{i}^{+}=\text{max}\left[0,\left(H_{i}-\mu_{z}\right)-\text{K}+MHC_{i-1}^{+}\right]\\ MHC_{i}^{-}=\text{max}\left[0,-\left(H_{i}-\mu_{z}\right)-\text{K}+MHC_{i-1}^{-}\right] \end{array}\right]$$ (2)

Where *H*_*i*_ is given in (1) and $MHC_{i}^{+}=MHC_{i}^{-}=0$. The K and *H* are defined as (cf. Abid et al. [15])

$$\text{K}=k\sqrt{Var\left(H_{i}\right)}=\begin{array}{ll} k\sqrt{\frac{\theta^{2}\sigma_{z}^{2}}{n}} & if\;i=1\\ k\sqrt{\frac{\theta^{2}\sigma_{z}^{2}}{n}+{\left(1-\theta^{2}\right)}^{2}\frac{\sigma_{z}^{2}}{n\left(i-1\right)}}, & \;if\;i>1 \end{array}]$$ (3)

$$\text{H}=h\sqrt{Var\left(H_{i}\right)}=\begin{array}{ll} h\sqrt{\frac{\theta^{2}\sigma_{z}^{2}}{n}} & if\;i=1\\ h\sqrt{\frac{\theta^{2}\sigma_{z}^{2}}{n}+{\left(1-\theta^{2}\right)}^{2}\frac{\sigma_{z}^{2}}{n\left(i-1\right)}}, & \;if\;i>1 \end{array}]$$ (4)

And these are the parameters of the MHC chart. The process is considered to be out-of-control (OOC) if any value of $MHC_{i}^{+}$ or $MHC_{i}^{-}$ go beyond *H*; otherwise, it is considered to be in control.

### 2.2. The proposed MHC_AIB_ chart

In most situations, there exists a positive/negative association between the study/process variable (*z*_*i*_) and the auxiliary variable(*x*_*i*_). Let us assume that *x*_*i*_ is strongly correlated with *z*_*i*_ and the strength of the correlation between *x*_*i*_ and *z*_*i*_ is represented by *ρ*_*zx*_. The pair of observations *x*_*i*_ and *z*_*i*_ follows the bivariate normal distribution, i.e., (*z*_*i*_, *x*_*i*_) ~ *BVN*(*μ*_*z*_ + *δσ*_*z*_, *μ*_*x*_, *σ*_*z*_, *σ*_*x*_, *ρ*_*zx*_), where *δ* is mathematically written as $\;\delta =\frac{\sqrt{n}}{\sigma_{z}}\left|\mu_{z}-\mu_{1}\right|$, where *μ*_1_ is the shifted mean. The regression estimator suggested by Cochran [30] is as follows:

$$R_{i}={\bar{z}}_{i}+b_{zx}\left(\mu_{x}-{\bar{x}}_{i}\right)$$ (5)

where $b_{zx}=\rho_{zx}\frac{\sigma_{z}}{\sigma_{x}}$ is the regression coefficient, ${\overset{-}{z}}_{i}$ and ${\overset{-}{x}}_{i}$ is the sample mean of the *z*_*i*_ and *x*_*i*_, respectively, and *μ*_*x*_ is the population mean of the *x*_*i*_. The mean and variance of *R*_*i*_ are given below (cf. Appendix A in S1 File):

$$\mu_{R}=\mu_{z}\;\text{and}\;\sigma_{R}^{2}=\;\frac{\sigma_{z}^{2}}{n}\left(1-\rho_{zx}^{2}\right),$$ (6)

The plotting statistic of the proposed MHC_AIB_ using (2) is defined as:

$$\left.\begin{array}{c} RHC_{i}^{+}=\text{max}\left[0,\left(RH_{i}-\mu_{z}\right)-\text{K}+RHC_{i-1}^{+}\right]\\ RHC_{i}^{-}=\text{max}\left[0,-\left(RH_{i}-\mu_{z}\right)-\text{K}+RHC_{i-1}^{-}\right] \end{array}\right]$$ (7)

Where

$$RH_{i}=\theta R_{i}+\;\left(1-\theta \right){\bar{R}}_{i-1}$$ (8)

*θ* is already defined above, $\;{\bar{R}}_{0}=\mu_{y},{\bar{R}}_{i-1}=\frac{\sum_{m=1}^{i-1}R_{m}}{i-1}$ and $RHC_{i}^{+}=RHC_{i}^{-}=0$. The MHC_AIB_ chart depends on two parameters, i.e., K and *H* and which are mathematically written as: (cf. Abid et al. [15]):

$$\text{K}=k\sqrt{Var\left(RH_{i}\right)}=\begin{array}{ll} k\sqrt{\frac{\theta^{2}}{n}\sigma_{z}^{2}\left(1-\rho_{zx}^{2}\right)} & if\;i=1\\ k\sqrt{\left(\frac{\theta^{2}}{n}+\frac{{\left(1-\theta^{2}\right)}^{2}}{n\left(i-1\right)}\right)\sigma_{z}^{2}\left(1-\rho_{zx}^{2}\right)} & \;if\;i>1 \end{array}]$$ (9)

$$\text{H}=h\sqrt{Var\left(RH_{i}\right)}=\begin{array}{ll} h\sqrt{\frac{\theta^{2}}{n}\sigma_{z}^{2}\left(1-\rho_{zx}^{2}\right)} & if\;i=1\\ h\sqrt{\left(\frac{\theta^{2}}{n}+\frac{{\left(1-\theta^{2}\right)}^{2}}{n\left(i-1\right)}\right)\sigma_{z}^{2}\left(1-\rho_{zx}^{2}\right)} & \;if\;i>1 \end{array}]$$ (10)

${RHC}_{i}^{+}$ and $R{HC}_{i}^{-}$ are plotted against the value of *H* given in (10). If ${RHC}_{i}^{+}>H$ or $R{HC}_{i}^{-}>H$, the process is assumed to be out of control (OOC); otherwise, it is in control (IC).

## 3. Performance evaluation of the MHC_AIB_ chart

**Fig 1:**
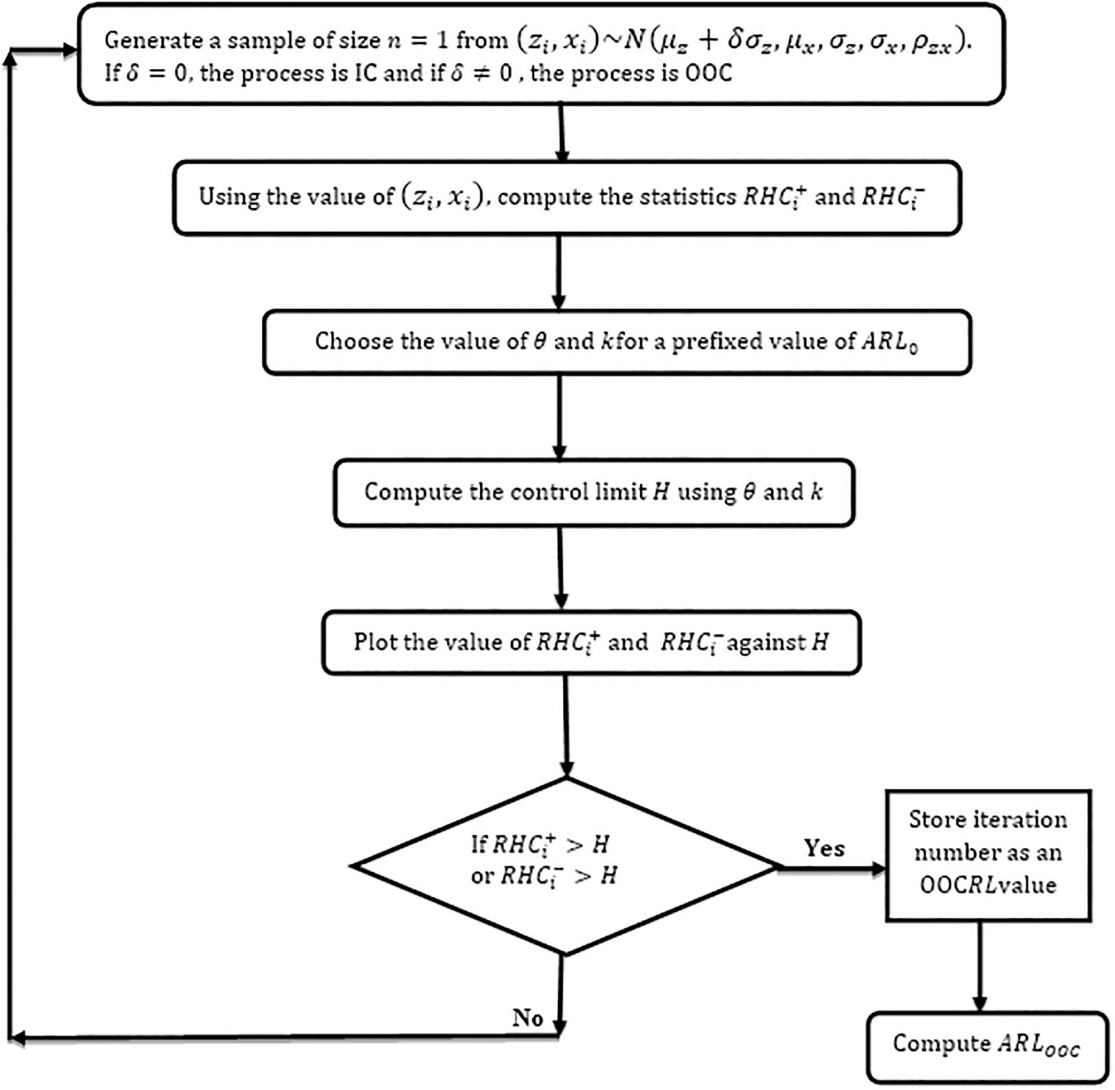
The computational algorithm of the proposed MHC_AIB_ chart.

The help of a well-known measure assesses the performance of the proposed MHCAIB and its competitor charts called the average run length (ARL). The Average Run length can be defined as “The number of sample points before a control chart gives alarm is called run length (RL) and an average value of RL distribution is called ARL.” The *ARL*_*IC*_ and *ARL*_*OOC*_ designated the in-control (IC) and out-of-control (OOC) ARL. A chart having a lesser value of *ARL*_*OOC*_ for a particular shift is designated to be better over the competitor chart at a certain shift in the process parameter(s) for a fixed value of *ARL*_*IC*_. We have also included some other performance measures associated with RL, like (the standard deviation RL (SDRL) and the median RL (MDRL) (cf. Abid et al. [15])) and these measures are calculated through the Monte Carlo simulations approach. The computational algorithms are developed in the R programming language, and the computational algorithms’ flow chart is presented in Fig 1.

The MHC_AIB_ chart has the design parameters *n*, *θ*, *ρ*_*zx*_, *h* and *k*. the *ARL*_*OOC*_ values of the proposed MHC_AIB_ chart against numerous choices of *θ* = 0.1, 0.25, 0.5, 0.75 and *ρ*_*zx*_ = 0.25, 0.5, 0.75, 0.95 when the value of *k* = 0.5 are given in Tables 1–4. The proposed MHC_AIB_ chart shows better performance against smaller values of *θ*(for instance *δ* = 0.1, *ρ*_*zx*_ = 0.25 when *θ* = 0.1, *ARL*_*OOC*_ = 103.54 against *θ* = 0.75, *ARL*_*OOC*_ = 300.25 (cf. Table 1 vs. Table 4)). An increase in *ρ*_*zx*_ enhanced the efficiency of the MHC_AIB_ chart (for instance *δ* = 0.05, *θ* = 0.1 when *ρ*_*zx*_ = 0.25, *ARL*_*OOC*_ = 117.43 against *ρ*_*zx*_ = 0.95, *ARL*_*OOC*_ = 39.74 (cf. Table 1)). There is a decrease in the OOC SDRL and MDRL values with the increase in *ρ*_*zx*_ (for instance *δ* = 0.125, *θ* = 0.1 when *ρ*_*zx*_ = 0.25, *OOC SDRL* = 71.08, OOC MDRL = 22 against *ρ*_*zx*_ = 0.95, *OOC SDRL* = 11.66, OOC MDRL = 12 (cf. Table 1)).

**Table 1:** ARL profiles of the proposed *MHC*_*AIB*_ chart for various choices of *ρ*_*zx*_ when *θ* = 0.10.

*δ*	*ρ*_*zx*_											
	0.25			0.5			0.75			0.95		
	ARL	SDRL	MDRL	ARL	SDRL	MDRL	ARL	SDRL	MDRL	ARL	SDRL	MDRL
0.05	117.43	330.51	26	107.81	283.32	26	81.51	174.14	26	39.74	51.08	21
0.075	79.94	170.53	26	71.00	141.90	25	55.88	91.46	24	26.49	26.75	17
0.1	60.77	103.54	24	53.52	83.82	24	41.03	55.58	21	19.98	16.85	14
0.125	48.51	71.08	22	43.52	59.43	22	33.67	38.87	19	16.01	11.66	12
0.15	41.10	52.68	22	37.92	49.11	21	27.52	27.53	18	13.36	8.68	11
0.175	35.35	42.97	20	31.33	37.19	19	23.90	22.44	16	11.64	6.85	10
0.2	30.63	33.86	19	26.87	28.15	17	21.38	19.15	15	10.19	5.45	9
0.25	24.90	24.31	16	22.18	19.80	15	17.10	13.03	13	8.42	3.70	7
0.5	12.44	7.64	10	11.20	6.37	9	8.82	4.01	8	5.06	1.20	5
0.75	8.73	4.00	8	7.91	3.28	7	6.42	2.11	6	4.00	0.69	4
1	6.93	2.48	6	6.37	2.05	6	5.26	1.34	5	3.41	0.52	3
1.5	5.17	1.30	5	4.83	1.07	5	4.13	0.73	4	3.01	0.09	3
2	4.36	0.84	4	4.09	0.72	4	3.53	0.56	4	2.88	0.34	3
***h***	**8.575**											

**Table 2:** ARL profiles of the proposed *MHC*_*AIB*_ chart for various choices of *ρ*_*zx*_ when *θ* = 0.25.

*δ*	*ρ*_*zx*_											
	0.25			0.5			0.75			0.95		
	ARL	SDRL	MDRL	ARL	SDRL	MDRL	ARL	SDRL	MDRL	ARL	SDRL	MDRL
0.05	262.14	578.16	35	229.30	490.45	36	169.30	341.59	33	56.88	86.91	23
0.075	162.23	321.10	33	140.22	272.26	31	93.42	166.15	28	32.15	38.09	18
0.1	110.16	201.02	28	93.41	160.70	28	63.36	101.08	24	21.60	21.46	14
0.125	77.61	132.10	26	67.04	106.21	25	44.52	60.35	21	16.15	13.88	11
0.15	60.21	92.09	24	51.56	74.87	22	33.92	42.41	18	12.87	9.66	10
0.175	48.25	69.14	21	40.13	52.29	20	27.69	31.96	16	10.84	7.19	9
0.2	40.05	54.03	20	33.76	41.19	18	23.35	24.30	15	9.33	5.57	8
0.25	28.71	33.30	17	24.84	26.76	15	17.40	15.68	12	7.56	3.79	7
0.5	12.06	8.82	9	10.46	6.82	8	7.99	4.30	7	4.30	1.12	4
0.75	7.78	4.05	7	6.97	3.22	6	5.54	2.01	5	3.36	0.57	3
1	6.00	2.42	5	5.46	1.99	5	4.45	1.23	4	3.01	0.32	3
1.5	4.40	1.21	4	4.06	0.99	4	3.46	0.63	3	2.40	0.51	2
2	3.64	0.75	4	3.43	0.61	3	3.06	0.33	3	1.78	0.44	2
***h***	**6.625**											

**Table 3:** ARL profiles of the proposed *MHC*_*AIB*_ chart for various choices of *ρ*_*zx*_ when *θ* = 0.5.

*δ*	*ρ*_*zx*_											
	0.25			0.5			0.75			0.95		
	ARL	SDRL	MDRL	ARL	SDRL	MDRL	ARL	SDRL	MDRL	ARL	SDRL	MDRL
0.05	370.80	521.37	161	359.00	493.57	158	292.51	408.95	130	116.32	151.77	57
0.075	290.12	402.43	128	261.84	351.97	124	190.26	249.90	91	57.40	68.82	32
0.1	221.19	298.70	103	187.51	248.80	89	126.50	161.65	64	33.70	35.31	21
0.125	156.20	205.41	75	135.08	173.55	68	87.53	108.64	46	22.93	21.24	16
0.15	123.84	160.76	62	100.98	125.41	53	62.99	75.14	35	16.97	14.19	12
0.175	94.60	119.63	48	77.40	93.43	42	48.38	54.90	28	13.33	10.19	10
0.2	73.09	86.36	40	61.04	72.44	34	36.64	39.07	22	10.94	7.57	9
0.25	50.02	57.60	29	41.24	44.91	25	25.54	24.71	17	8.16	4.74	7
0.5	14.98	12.03	11	12.90	9.79	10	8.74	5.25	7	3.98	1.22	4
0.75	8.46	4.97	7	7.48	4.09	6	5.51	2.34	5	2.95	0.63	3
1	6.04	2.83	5	5.40	2.26	5	4.15	1.32	4	2.40	0.51	2
1.5	4.09	1.31	4	3.72	1.05	3	3.06	0.67	3	1.90	0.33	2
2	3.26	0.76	3	3.02	0.65	3	2.51	0.53	2	1.36	0.48	1
***h***	**5.536**											

**Table 4:** ARL profiles of the proposed *MHC*_*AIB*_ chart for various choices of *ρ*_*zx*_ when *θ* = 0.75.

*δ*	*ρ*_*zx*_											
	0.25			0.5			0.75			0.95		
	ARL	SDRL	MDRL	ARL	SDRL	MDRL	ARL	SDRL	MDRL	ARL	SDRL	MDRL
0.05	430.62	457.84	284.5	415.16	442.92	276	371.10	407.80	240	190.93	200.22	126
0.075	372.53	405.59	239	350.00	371.77	232.5	279.72	304.95	180	100.69	103.63	67
0.1	300.25	326.22	191	272.43	292.73	179	203.04	217.61	131	58.76	56.94	40
0.125	242.70	262.71	158	213.69	230.35	137.5	149.28	159.03	97	37.43	34.60	26
0.15	199.04	208.87	128	169.69	181.26	110	110.07	118.21	71	26.25	22.43	19
0.175	162.29	171.48	105.5	132.78	139.83	88	82.65	83.97	56	19.62	15.02	15
0.2	128.11	137.00	83	106.64	110.47	70	65.93	66.42	44	15.48	11.20	12
0.25	88.92	90.87	59	71.37	71.88	47	41.77	39.33	29	10.73	6.46	9
0.5	22.51	18.08	17	18.40	14.13	14	11.84	7.59	10	4.36	1.53	4
0.75	11.29	6.94	9	9.54	5.37	8	6.61	3.09	6	2.92	0.77	3
1	7.47	3.64	7	6.47	2.99	6	4.65	1.72	4	2.29	0.49	2
1.5	4.54	1.66	4	4.01	1.33	4	3.06	0.83	3	1.82	0.39	2
2	3.36	0.98	3	3.05	0.82	3	2.40	0.55	2	1.24	0.43	1
***h***	**5.187**											

**Fig 2:**
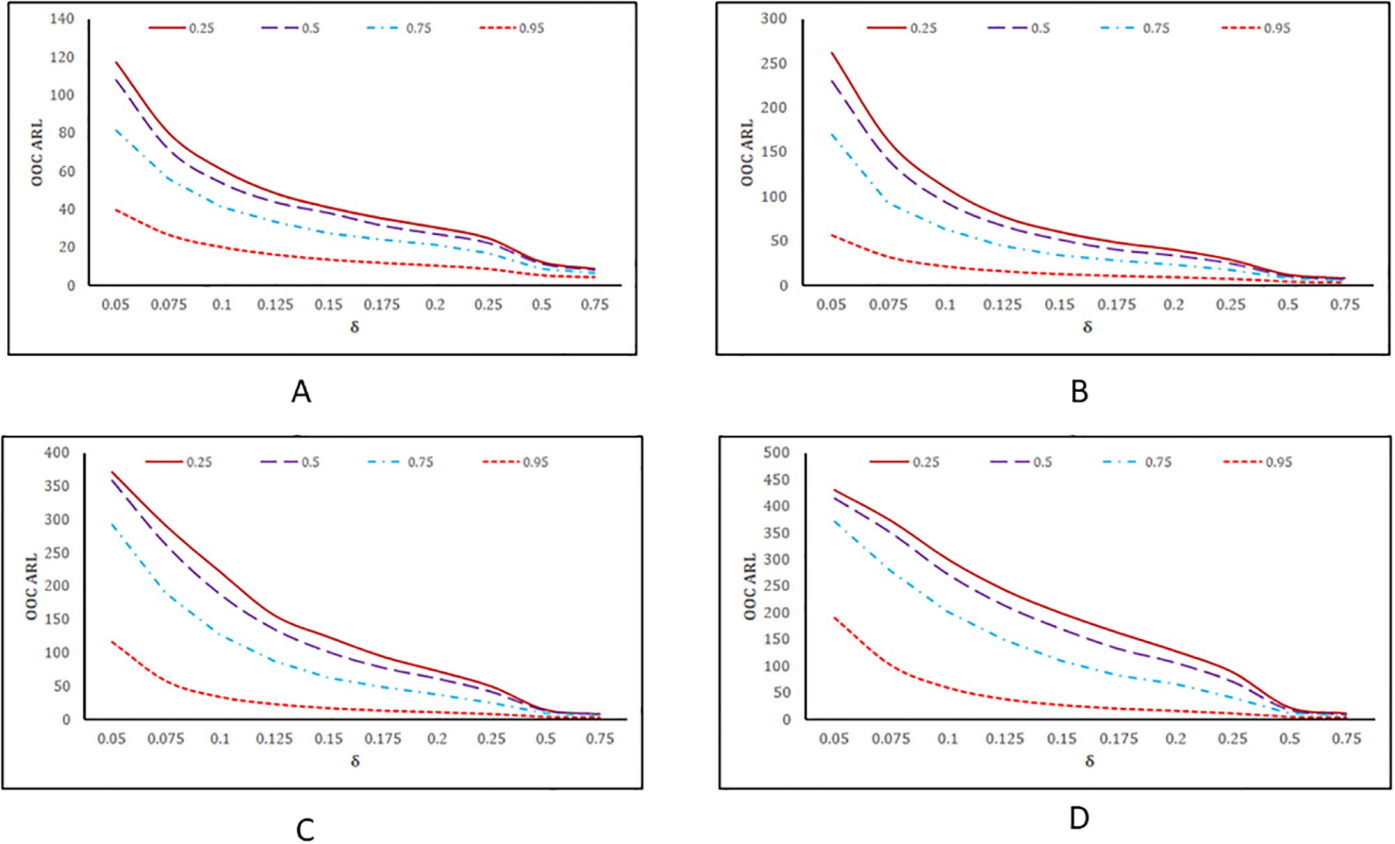
*ARL*_*OOC*_ values based graphical comparison of the proposed MHC_AIB_ chart for various choices of *ρ*_*zx*_. (A). when *θ* = 0.1, (B). when *θ* = 0.25, (C). when *θ* = 0.5 and (D) when *θ* = 0.75.

The performance assessment of the MHC_AIB_ chart in the form of a line graph is given in Fig 2A–2D against various choices of *θ* and *ρ*_*zx*_. A decrease in *θ* enhanced the efficacy of the MHC_AIB_ chart and vice versa (cf. Fig 2A vs. Fig 2D). Also, an increase in the value of *ρ*_*zx*_ improved the sensitivity of the MHC_AIB_ chart and vice versa. It means that a higher correlation coefficient value increases the suggested chart’s efficiency. (cf. Fig 2A–2D).

### 3.1. Comparisons

This section offered the OOC performance assessment of the proposed MHC_AIB_ with the CUSUM_AIB_, EWMA_AIB_, HWMA_AIB_, and MEC_AIB_ suggested by Abbas et al. [18], Abbas [19], Sanusi et al. [22], and Anwar et al. [27] respectively. Moreover, we have also expressed these comparisons as a percentage decrease in ARL. (*ARL*_*PD*_) and mathematically *ARL*_*PD*_ is defined as $\left(\frac{{ARL}_{IC}-{ARL}_{OOC}}{{ARL}_{IC}}\right)\times 100\%$. The chart with the highest *ARL*_*PD*_ value is labeled an efficient chart for that specific shift.

#### 3.1.1. MHC_AIB_ versus CUSUM_AIB_

Abbas et al. [18] suggested the CUSUM_AIB_ chart and the *ARL*_*OOC*_ results of the CUSUM_AIB_ chart are provided in Tables 5 and 6 against various choices of *θ*, *ρ*_*zx*_ and *δ*. The proposed MHC_AIB_ chart compromises enhanced performance over the CUSUM_AIB_ chart for all selections of *θ*, *ρ*_*zx*_, and *δ* (for instance when *δ* = (0.05, 0.1, 0.2), *θ* = 0.1 and *ρ*_*zx*_ = 0.5, the MHC_AIB_ *ARL*_*OOC*_ = (108, 54, 27) and the CUSUM_AIB_ *ARL*_*OOC*_ = (374, 220, 92) (cf. Table 5). Also, at *δ* = 0.05, the *ARL*_*PD*_ in CUSUM_AIB_ and MHC_AIB_ charts are 25.2%and 78.4%, when *θ* = 0.1 and *ρ*_*zx*_ = 0.5, respectively.

**Table 5:** ARL comparisons between proposed MHC_AIB_ and existing charts for various choices of ρ_zx_ when θ = 0.1.

*δ*	CUSUM_AIB_			EWMA_AIB_			HWMA_AIB_			MEC_AIB_			MHC_AIB_		
	*ρ*_*zx*_			*ρ*_*zx*_			*ρ*_*zx*_			*ρ*_*zx*_			*ρ*_*zx*_		
	0.5	0.75	0.95	0.5	0.75	0.95	0.5	0.75	0.95	0.5	0.75	0.95	0.5	0.75	0.95
0.0	498	502	501	501	504	499	502	502	502	499	502	500	500	501	500
0.05	374	320	150	421	378	200	374	315	149	370	303	143	108	82	40
0.075	287	223	88	350	288	111	286	222	87	275	213	84	71	56	26
0.1	220	161	60	282	214	67	216	160	57	212	153	58	54	41	20
0.125	170	122	45	228	160	44	168	120	40	162	114	45	44	34	16
0.15	135	95	36	182	121	31	135	94	31	128	91	37	38	28	13
0.175	111	78	30	145	94	23	110	75	24	106	75	31	31	24	12
0.2	92	65	26	118	74	18	91	62	19	88	62	28	27	21	10
0.25	69	49	20	81	49	12	66	44	13	66	48	23	22	17	8
0.5	29	22	10	22	13	4	23	15	5	31	24	13	11	9	5
0.75	18	14	6	11	7	2	12	8	3	21	17	10	8	6	4
1	14	10	5	6	4	1	7	5	2	17	14	8	6	5	3
1.5	9	7	3	3	2	1	4	3	1	12	10	6	5	4	3
2	7	5	3	2	2	1	3	2	1	10	8	5	4	4	3
	***k* = 0.5, *h* = 5.071**			***C* = 2.824**			***C* = 2.936**			***k* = 0.5, *h* = 37.35**			***k* = 0.5, *h* = 8.575**		

**Table 6:** ARL comparisons between proposed MHC_AIB_ and existing charts for various choices of ρ_zx_ when θ = 0.25.

*δ*	CUSUM_AIB_			EWMA_AIB_			HWMA_AIB_			MEC_AIB_			MHC_AIB_		
	*ρ*_*zx*_			*ρ*_*zx*_			*ρ*_*zx*_			*ρ*_*zx*_			*ρ*_*zx*_		
	0.5	0.75	0.95	0.5	0.75	0.95	0.5	0.75	0.95	0.5	0.75	0.95	0.5	0.75	0.95
0.0	498	502	501	499	500	500	501	500	501	502	499	500	499	500	499
0.05	374	320	150	455	429	285	425	385	209	396	337	161	229	169	57
0.075	287	223	88	414	369	179	355	296	120	307	241	89	140	93	32
0.1	220	161	60	361	299	114	290	224	75	238	173	58	93	63	22
0.125	170	122	45	311	242	75	235	171	51	187	128	42	67	45	16
0.15	135	95	36	265	194	52	191	132	36	145	97	33	52	34	13
0.175	111	78	30	225	157	37	154	104	27	116	77	27	40	28	11
0.2	92	65	26	190	127	27	128	83	22	96	64	23	34	23	9
0.25	69	49	20	137	84	17	90	56	14	68	46	18	25	17	8
0.5	29	22	10	35	19	4	26	16	4	26	19	9	10	8	4
0.75	18	14	6	14	8	2	13	8	2	16	12	7	7	6	3
1	14	10	5	8	5	1	8	5	2	12	10	5	5	4	3
1.5	9	7	3	4	2	1	4	3	1	9	7	4	4	3	2
2	7	5	3	2	2	1	3	2	1	7	6	3	3	3	2
	***k* = 0.5, *h* = 5.071**			***C* = 3.001**			***C* = 3.075**			***k* = 0.5, *h* = 20.17**			***k* = 0.5, *h* = 6.625**		

#### 3.1.2. MHC_AIB_ versus EWMA_AIB_

Abbas [19] recommended the EWMA_AIB_ chart and the results of *ARL*_*OOC*_ values of the EWMA_AIB_ chart are specified in Tables 5 and 6. The suggested MHC_AIB_ chart displays comparatively better performance over the EWMA_AIB_ chart when *δ* ≤ 0.75 (for instance, at *δ* = (0.05, 0.1, 0.2), *θ* = 0.1, and *ρ*_*zx*_ = 0.5, the MHC_AIB_ *ARL*_*OOC*_ = (108, 54, 27) and the EWMA_AIB_ *ARL*_*OOC*_ = (421, 282, 118) (cf. Table 5) and when *θ* = 0.25, the MHC_AIB_ *ARL*_*OOC*_ = (229, 93, 34) and the EWMA_AIB_ *ARL*_*OOC*_ = (455, 361, 190) (cf. Table 6). Furthermore, at *δ* = 0.1, the *ARL*_*PD*_ in AIB-EWMA and AIB-MHC charts are 43.6% and 89.2% when *θ* = 0.1 and *ρ*_*zx*_ = 0.5, respectively.

#### 3.1.3. MHC_AIB_ versus HWMA_AIB_

Sanusi et al. [22] introduced the HWMA_AIB_ chart and the results of *ARL*_*OOC*_ values of HWMA_AIB_ chart are provided in Tables 5 and 6 for several choices of *θ*, *ρ*_*zx*_, and *δ*. The suggested MHC_AIB_ chart shows a better *ARL*_*OOC*_ performance against the HWMA_AIB_ chart for all choices of *θ* and *ρ*_*zx*_ when *δ* ≤ 0.75 (for instance at *δ* = (0.05, 0.1, 0.2), *θ* = 0.1, and *ρ*_*zx*_ = 0.75, the MHC_AIB_ *ARL*_*OOC*_ = (82, 41, 21) and the HWMA_AIB_ *ARL*_*OOC*_ = (315, 160, 62) (cf. Table 5) and when *θ* = 0.25, the MHC_AIB_ *ARL*_*OOC*_ = (169, 63, 23) and the HWMA_AIB_ *ARL*_*OOC*_ = (385, 224, 83), (cf. Table 6). Moreover, at *δ* = 0.1, the *ARL*_*PD*_ in HWMA_AIB_ and MHC_AIB_ charts are 55.2% and 87.4% when *θ* = 0.25 and *ρ*_*zx*_ = 0.75, respectively.

#### 3.1.4. MHC_AIB_ versus MEC_AIB_

Anwar et al. [29] suggested the MEC_AIB_ chart and the results of *ARL*_*OOC*_ values of MEC_AIB_ chart are specified in Tables 5 and 6. The suggested MHC_AIB_ chart proposes reasonably superior performance against the MEC_AIB_ chart for all choices of *θ*, *ρ*_*zx*_ and *δ* (for instance at *δ* = 0.05, 0.1, 0.2, *θ* = 0.1 and *ρ*_*zx*_ = 0.75, the MHC_AIB_ *ARL*_*OOC*_ = (82, 41, 21) and the MEC_AIB_ *ARL*_*OOC*_ = (303, 153, 62) (cf. Table 5) and when *θ* = 0.25, the MHC_AIB_ *ARL*_*OOC*_ = (169, 63, 23) and the MEC_AIB_ *ARL*_*OOC*_ = (337, 173, 64) (cf. Table 6). Also, at *δ* = 0.1, the *ARL*_*PD*_ in AIB-MEC and AIB-MHC charts are 65.4% and 87.4% when *θ* = 0.25 and *ρ*_*zx*_ = 0.75, respectively.

### 3.2. Graphical comparisons based on *ARL*_*OOC*_

**Fig 3:**
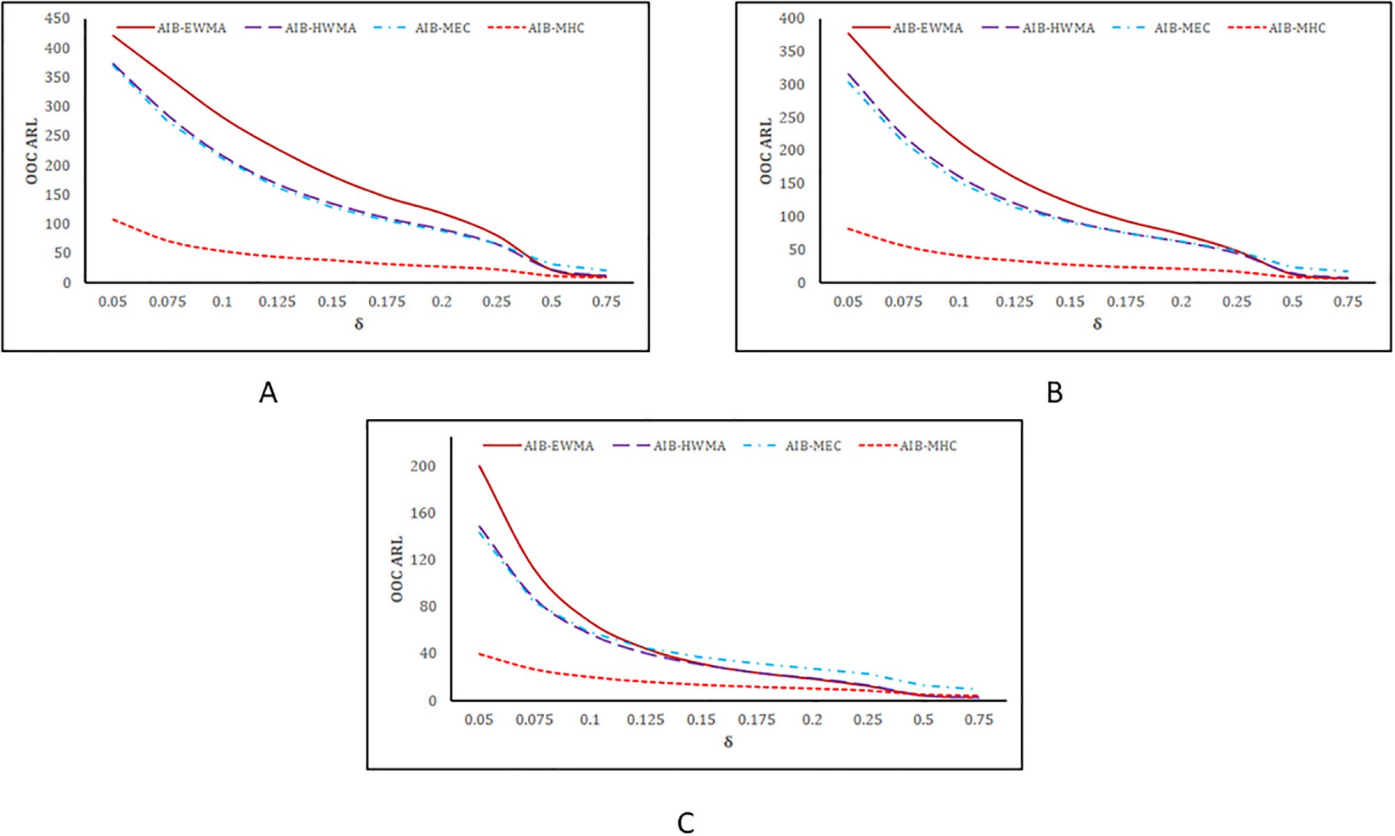
*ARL*_*OOC*_ values based graphical comparison between the proposed MHC_AIB_ and existing charts for *θ* = 0.1. (A) when *ρ*_*zx*_ = 0.5, (B) when *ρ*_*zx*_ = 0.75, (C) when when *ρ*_*zx*_ = 0.95.

The *ARL*_*OOC*_ based graphical comparisons of the proposed MHC_AIB_, EWMA_AIB_, HWMA_AIB_, and MEC_AIB_ charts are presented in Fig 3A–3C for various choices *ρ*_*zx*_. The proposed MHC_AIB_ chart offers inferior performance for the selected choices of *θ* and *ρ*_*zx*_ (cf. Fig 3A–3C). Additionally, the proposed MHC_AIB_ chart displays superiority over the EWMA_AIB_, HWMA_AIB_, and MEC_AIB_ charts as the value of *ρ*_*zx*_ is increased (cf. Fig 3A vs. Fig 3D).

## 4. An illustrative example

Based on the simulated dataset, this section offers an illustrative example of the proposed MHCAIB, HWMAAIB, and MECAIB charts. This dataset consists of 20 pairs of observations which are obtained from the bivariate normal distribution, i.e., (*z*_*i*_, *x*_*i*_) ~ *N*(*μ*_*z*_ + *δσ*_*z*_, *μ*_*x*_, *σ*_*z*_, *σ*_*x*_, *ρ*_*zx*_) by using the following values of *μ*_*z*_ = 0, *δ* = 0.50, *μ*_*x*_ = 0, *σ*_*z*_ = 1, *σ*_*x*_ = 1 and *ρ*_*zx*_ = 0.50 (cf. Abbas et al. [18]). This dataset is used to evaluate the shift-detecting capability of the proposed MHC_AIB_, HWMA_AIB_, and MEC_AIB_ charts. The selected parameters for the practical implementation of the proposed MHC_AIB_, HWMA_AIB_, and MEC_AIB_ charts are as follows: for the proposed MHC_AIB_ chart *θ* = 0.1, *k* = 0.5, and *h* = 8.575; for the MEC_AIB_ chart *θ* = 0.1, *k* = 0.5, and *h* = 37.35; for HWMA_AIB_ chart *θ* = 0.1, and *C* = 2.936 when *ARL*_*IC*_ ≈ 500. The control limits and the plotting statistics of the MHC_AIB_, HWMA_AIB_, and MEC_AIB_ charts are given in Table 7.

**Table 7:** The plotting-statistic and the control limits values of the proposed MHC_AIB_, MEC_AIB_, and HWMA_AIB_ charts.

*i*	*z*_*i*_	*x*_*i*_	HWMA_AIB_ chart			MEC_AIB_ chart			MHC_AIB_ chart		
			HWMA_AIB_	UCL	LCL	*MEC* ^+^	*MEC* ^−^	*H*	*RHC* ^+^	*RHC* ^−^	*H*
1	0.39	-0.865	0.082	0.254	-0.254	0.039	0	3.235	0.039	0	0.743
2	-0.242	-1.686	0.800	2.304	-2.304	0.115	0	4.352	0.447	0	6.725
3	-0.919	-1.046	0.601	1.639	-1.639	0.128	0	5.080	0.769	0	4.784
4	-1.22	-1.366	0.255	1.346	-1.346	0.072	0	5.600	0.795	0	3.930
5	2.01	0.574	0.283	1.173	-1.173	0.182	0	5.989	0.878	0	3.423
6	1.395	1.61	0.457	1.055	-1.055	0.327	0	6.286	1.156	0	3.080
7	1.66	1.542	0.509	0.969	-0.969	0.536	0	6.517	1.500	0	2.828
8	-0.514	0.816	0.383	0.902	-0.902	0.620	0	6.698	1.729	0	2.633
9	-0.213	-0.907	0.336	0.849	-0.849	0.709	0	6.841	1.920	0	2.477
10	-0.588	-1.923	0.338	0.805	-0.805	0.816	0	6.955	2.122	0	2.348
11	0.074	0.132	0.305	0.768	-0.768	0.902	0	7.046	2.297	0	2.240
12	1.673	1.64	0.363	0.736	-0.736	1.055	0	7.119	2.534	0	2.148
13	1.765	0.575	0.466	0.708	-0.708	1.329	0	7.177	2.880	0	2.067
14	0.061	-0.008	0.403	0.684	-0.684	1.573	0	7.224	3.166	0	1.997
15	1.537	-1.084	0.580	0.663	-0.663	1.990	0	7.262	3.633	0	1.934
16	-0.519	-0.52	0.446	0.644	-0.644	2.329	0	7.292	3.970	0	1.879
17	1.198	-0.246	0.560	0.626	-0.626	2.756	0	7.317	4.423	0	1.828
18	1.853	0.028	0.657	0.611	-0.611	3.315	0	7.337	4.976	0	1.783
19	0.733	1.715	0.526	0.597	-0.597	3.795	0	7.353	5.400	0	1.742
20	0.108	0.6	0.545	0.584	-0.584	4.198	0	7.366	5.786	0	1.704

**Fig 4:**
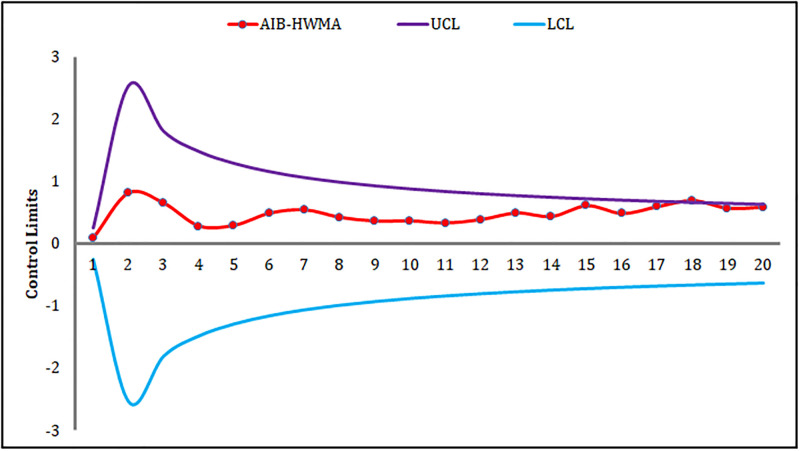
An application of the HWMA_AIB_ chart.

**Fig 5:**
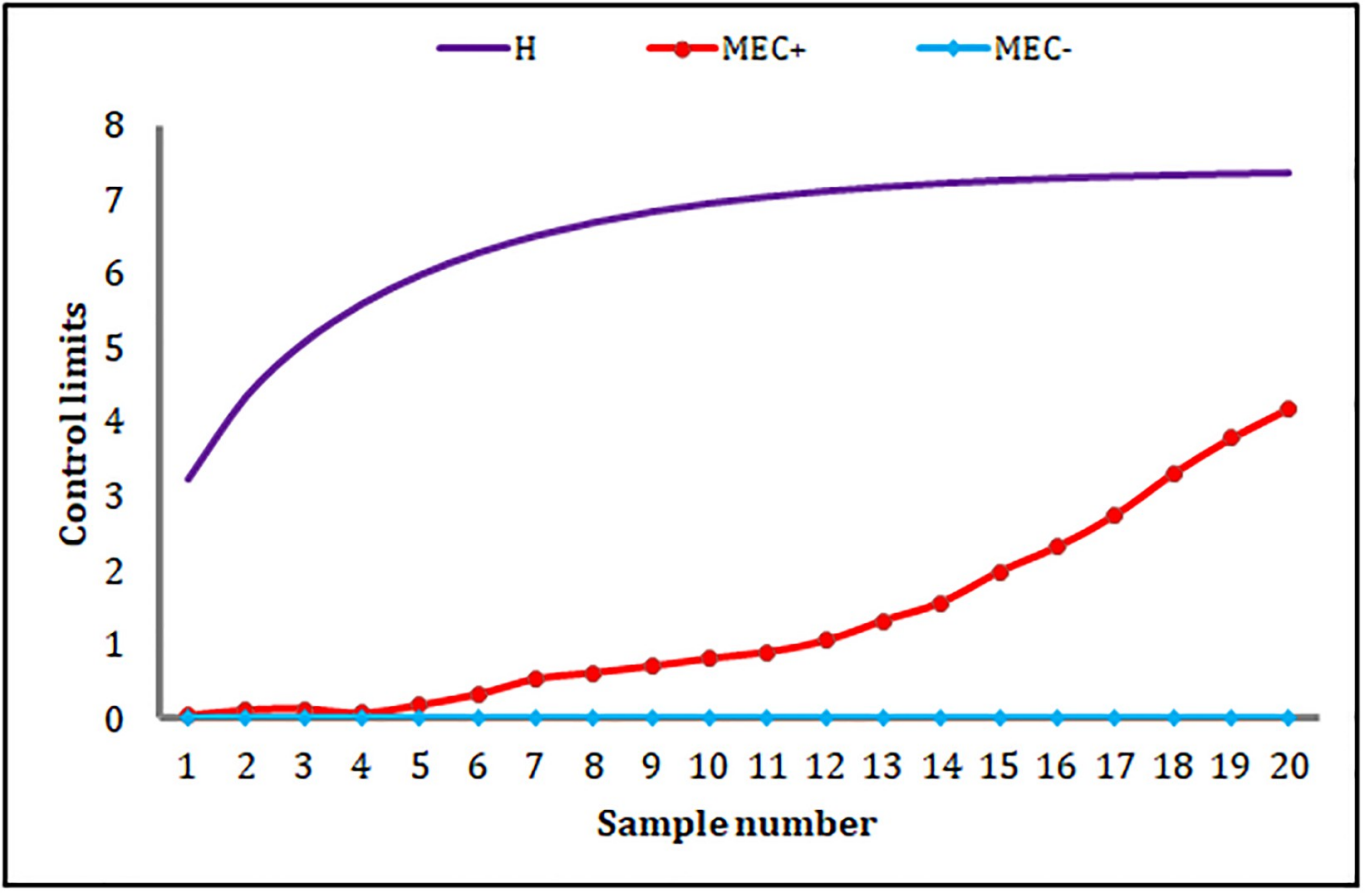
An application of the MEC_AIB_ chart.

**Fig 6:**
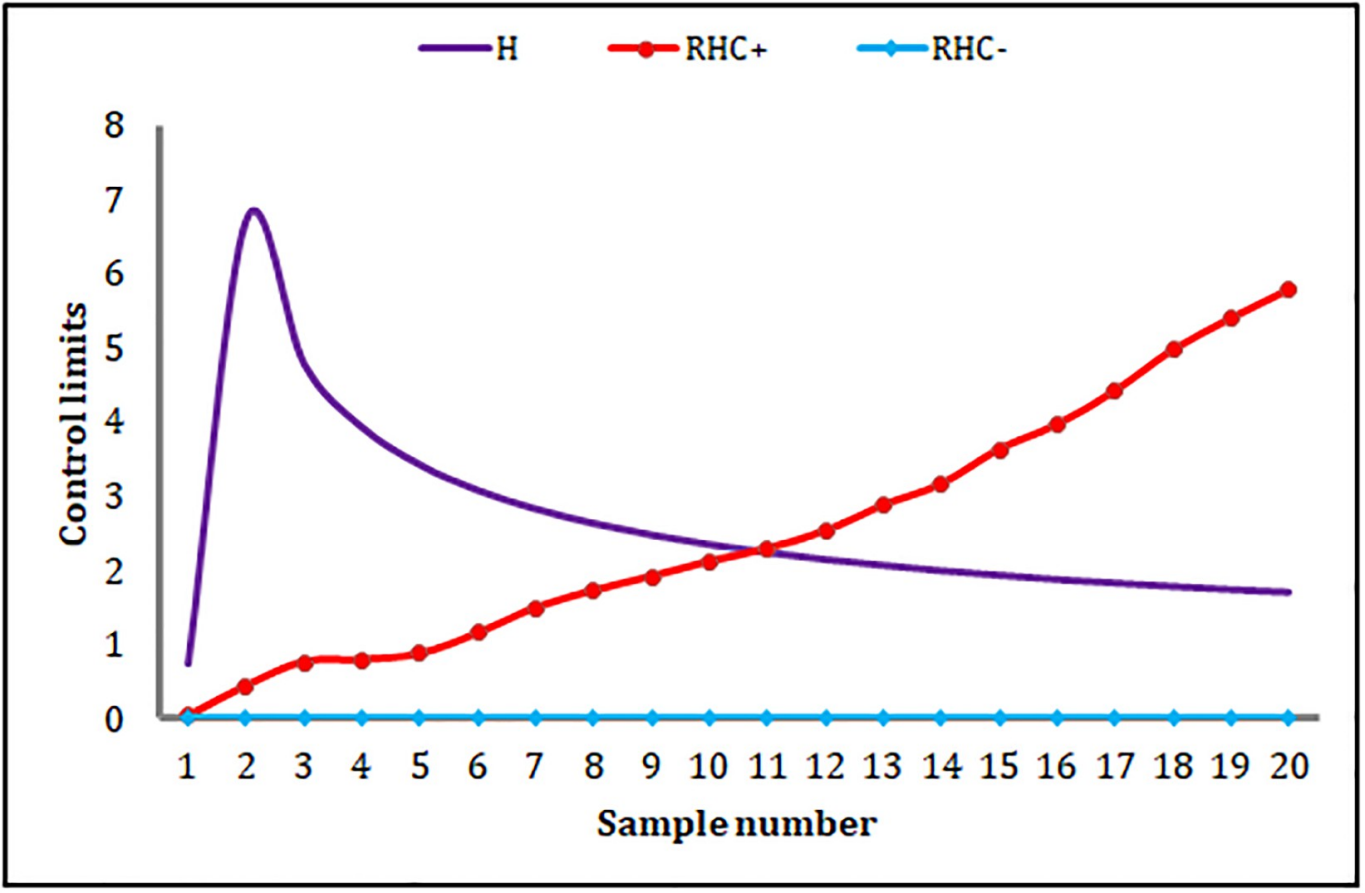
An application of the MHC_AIB_ chart.

The HWMA_AIB_ chart signals only one OOC point at the 18^th^ sample (cf. Fig 4). The MEC_AIB_ chart cannot produce any OOC signal (cf. Fig 5). Moreover, the proposed MHC_AIB_ chart produces nine OOC signals from sample numbers 12 to 20 (cf. Fig 6), and this is a piece of evidence about the enhanced shift-detecting capability of the proposed MHC_AIB_ chart against the HWMA_AIB_, and MEC_AIB_ charts.

## 5. Limitation

The proposed chart uses auxiliary information in its design, so it should be used only if there is a high correlation between the auxiliary variable and the study variable.

## 6. Conclusion and recommendations

A control chart is the most famous statistical process control and monitoring tool to detect irregular variations from ongoing processes. In this article, we have suggested a new regression estimator-based MHC chart labeled MHC_AIB_ for monitoring persistent deviations in the process location. The *ARL*_*OOC*_ performance of the suggested MHC_AIB_ chart is compared with the CUSUM_AIB_, EWMA_AIB_, HWMA_AIB_, and MEC_AIB_, and the suggested MHC_AIB_ chart performs exceptionally well in detecting shifts over its competitor charts for all selected sets of *θ* and *ρ*_*zx*_. Also, it is noticed that the choice of the larger value of *ρ*_*zx*_ and a smaller value of *θ* is effective in enhancing the performance of the MHC_AIB_ chart. An application based on simulated data has also identified the dominance of the MHC_AIB_ chart against the HWMA_AIB_ and MEC_AIB_ charts.

This study can also be extended for dual auxiliary information-based regression estimator for detecting deviations in the process location and dispersion.

## Supporting information

S1 File (DOCX)
