# Supplementary material for: Enhanced performance of mixed HWMA-CUSUM charts using auxiliary information
Source: PLoS One. 2023 Sep 15;18(9):e0290727. doi: 10.1371/journal.pone.0290727 (PMC10503744; doi:10.1371/journal.pone.0290727)
Supplement: S1 File — (DOCX) [file pone.0290727.s001.docx]

**Appendix-A**

The regression estimator used to estimate the process mean $\mu_{z}$ ids are written as:

$$R_{i}=\bar{z}_{i}+b_{zx}\left( \mu_{x}-\bar{x}_{i} \right)$$

Where, $b_{zx}=\rho_{zx}\frac{\sigma_{z}}{\sigma_{x}},$ shows the average change in $z$ when there is a unit change in $x.$

**Appendix-A1**

The IC mean of$R_{i}$ is derived as:

$$E\left( R_{i} \right)=E\left[ \bar{z}_{i}+b_{zx}\left( \mu_{x}-\bar{x}_{i} \right) \right],$$

$$E\left( R_{i} \right)=E\left( \bar{z}_{i} \right)+b_{zx}\left( \mu_{x}-E(\bar{x}_{i}) \right),$$

$E\left( R_{i} \right)=\mu_{z}+b_{zx}\left( \mu_{x}-\mu_{x} \right)$ , because $E\left( \bar{z}_{i} \right)=\mu_{z}$ and $E\left( \bar{x}_{i} \right)=\mu_{x}$

$$E\left( R_{i} \right)=\mu_{z}.$$

**Appendix-A2**

The IC variance of $R_{i}$ is derived as:

$Var\left( R_{i} \right)={E[R_{i}-E\left( R_{i} \right)]}^{2},$

$Var\left( R_{i} \right)=E{[\bar{z}_{i}+b_{zx}\left( \mu_{x}-\bar{x}_{i} \right)-\mu_{z}]}^{2}$ (cf. Appendix-A1)

$$Var\left( R_{i} \right)=E{[\left( \bar{z}_{i}-\mu_{z} \right)-b_{zx}\left( \bar{x}_{i}-\mu_{x} \right)]}^{2},$$

$$Var\left( R_{i} \right)=E\left[ \left( \bar{z}_{i}-\mu_{z} \right)^{2}+b_{zx}^{2}\left( \bar{x}_{i}-\mu_{x} \right)^{2}-2b_{zx}\left( \bar{z}_{i}-\mu_{z} \right)\left( \bar{x}_{i}-\mu_{x} \right) \right],$$

$$Var\left( R_{i} \right)=Var\left( \bar{z}_{i} \right)+b_{zx}^{2}Var\left( \bar{x}_{i} \right)-2b_{zx}Cov\left( \bar{z}_{i},\bar{x}_{i} \right),$$

$Var\left( R_{i} \right)=\frac{\sigma_{z}^{2}}{n}+b_{zx}^{2}\frac{\sigma_{x}^{2}}{n}-2b_{zx}\frac{\sigma_{zx}}{n}$ (A2-1)

Because$Var\left( \bar{z}_{i} \right)=\frac{\sigma_{z}^{2}}{n}, Var\left( \bar{x}_{i} \right)=\frac{\sigma_{x}^{2}}{n}$, and $Cov\left( \bar{z}_{i},\bar{x}_{i} \right)=\frac{\sigma_{zx}}{n}$

As $\rho_{zx}=\frac{\sigma_{zx}}{\sigma_{z}\times\sigma_{x}} \gg\sigma_{zx}=\rho_{zx}\sigma_{z}\sigma_{x}$ and

Also,$b_{zx}=\rho_{zx}\left( \frac{\sigma_{z}}{\sigma_{x}} \right)$

After putting the value of $\sigma_{zx}$ and $b_{zx}$ in (A2-1), we get

$$Var\left( R_{i} \right)=\frac{\sigma_{z}^{2}}{n}+\rho_{zx}^{2}\left( \frac{\sigma_{z}}{\sigma_{x}} \right)^{2}\times\frac{\sigma_{x}^{2}}{n}-2\rho_{zx}\left( \frac{\sigma_{z}}{\sigma_{x}} \right)\times\frac{\rho_{zx}\sigma_{z}\sigma_{x}}{n},$$

$$Var\left( R_{i} \right)=\frac{\sigma_{z}^{2}}{n}-\rho_{zx}^{2}\times\frac{\sigma_{z}^{2}}{n}$$

$Var\left( R_{i} \right)=\frac{\sigma_{z}^{2}}{n}\left( 1-\rho_{zx}^{2} \right)$.
