# Supplementary material for: Enhanced performance of mixed HWMA-CUSUM charts using auxiliary information
Source: PLoS One. 2023 Sep 15;18(9):e0290727. doi: 10.1371/journal.pone.0290727 (PMC10503744; doi:10.1371/journal.pone.0290727)
Supplement: Attachment — Submitted filename: Report.pdf [file pone.0290727.s002.pdf]

Report

General comments

In this manuscript, the performance of the mixed homogeneously weighted moving average (HWMA) cumulative sum (CUSUM) control chart is improved using the auxiliary information based (AIB) regression estimator, denoted as MHCAIB. The proposed MHCAIB chart is provided. The performance of the proposed MHCAIB is evaluated in terms of the run length properties and the proposed chart is compared with existing AIB charts like CUSUMAIB, EWMAAIB, MECAIB (mixed AIB EWMA-CUSUM), and HWMAAIB. The RL based performance comparisons indicate that the MHCAIB chart achieves relatively better performance to monitor small to moderate shifts over its competitors. The manuscript is not well-written and it is full of mistake. **My recommendation is to reject it**.

Major comments:

1. The manuscript is not well-written. Authors should consider to use an English language editor to improve the writing.
2. The motivation of this manuscript is very week. You cannot introduce a control chart because others introduced similar charts. Moreover, it is stated in the literature that the HWMA chart performs better for small shifts in the process parameter. Is there any need to combined it with the CUSUM and afterwards use an AIB to improve it again? What do you want to achieve? Did you consider the complexity of the resulting chart?
3. It looks like the authors did not check the formulas properly. There are many mistakes. Below Equation (1) what do $\bar{y}_m$ and t? I think they were used by mistake. Check other equations and derivations as well.
4. Knoth et al. (2021) advised against using the HWMA chart. They stated that the HWMA chart performs worst in steady-state and it is not recommended in practice. The same authors advised against the use of AIB chart in another article. Since both procedures are criticised, do you think that the resulting chart will be acceptable? Elaborate on this.
5. I recommend the investigation of the proposed MHC_{AIB} chart in terms of the conditional expected delay (CED) and compare it to its counterparts considered in this manuscript in terms of the CED properties.

Minor comments:

6. The first time an acronym is used, it must be defined fully- see for instance, RL in the abstract.
7. In the introduction, page 8, add a space between Shewhart and was. Check the entire manuscript to fix similar issues and add space where required.
8. Provide more recent citations
9. Fix the punctuation issues throughout the manuscript.
10. The list of mistakes is very long reason why I did not include it in the report.
