# Supplementary material for: Enhanced performance of mixed HWMA-CUSUM charts using auxiliary information
Source: PLoS One. 2023 Sep 15;18(9):e0290727. doi: 10.1371/journal.pone.0290727 (PMC10503744; doi:10.1371/journal.pone.0290727)
Supplement: Attachment — Submitted filename: response to the reviewers.docx [file pone.0290727.s003.docx]

**Response to Reviewers Comments on Article.**

**1^st^ Reviewers Comments**

1. **Design is good and study is Significant**
2. **Some new high quality methods should be added to the literature : “**We have added some new studies relevant to high quality methods”
3. **Some recent Articles are to be added to the literature based on Auxiliary Information: “**Recent studies in year 2023 on the use of HWMA and AIB information” have been added
4. **Add limitations:** We have added the limitations to the revised article

**2^nd^ Reviewer’s Comments**

1. **Provide Comparisons based on zero state and ready State:** with in the recent frame work of the study comparisons based on zero state and ready state cannot be done but we are working on this idea and will present this in some other article. However your suggestion is highly acknowledged.
2. **Provide detailed comparison with other HWMA types**: in this study we are focusing at the improvements in the design using the auxiliary information so we made comparisons with the existing counterparts which use auxiliary information.

**3^rd^ Reviewer’s Comments**

1. **Consider Use of English language editor to improve language:** complying with your kind suggestion we have improved the language of the article and corrected the mistakes.
2. **Motivation is weak:**

In some industrial and manufacturing processes auxiliary information is also recorded along with the information on the under study variables for different other tasks. This information can be used to improve the design of control chart without imparting any additional financial burden to the entrepreneur. We can use this information for the improvement in design.

Also in high quality monitoring a technique which provides better quality control is required because in the rapidly changing world quality is actually the other name of customers’ satisfaction.

Our proposed chart can be used for high quality monitoring in different industrial applications.

3. **Some formulae are not well written:** we have made corrections to the formulae and other typing mistakes.
4. **Knoth (2021) advised against the use of HWMA and auxiliary information.**

Abbas (2022) answered the objection related to use of HWMA for steady states applications. He suggested that a careful choice of HWMA parameter can provide good results even for the steady state. So HWMA can be applied with a careful choice of parameter

Also Knoth’s objection related to the use of auxiliary information is to use it when the auxiliary information is highly correlated. So as per definition of auxiliary information it should be correlated with the study variable, we also suggest the use of the proposed design only when there is a high correlation between the under study variable and the auxiliary variable.

It is also shown that the efficiency of the design improves with the increase in the value of correlation coefficient.

5. **Recommended the study in term of Conditional expected delay.** Thanks for your kind suggestion. We have started working on this idea and will be presented in future work.

**Thanks and Regards.**
