# Supplementary material for: Enhanced performance of mixed HWMA-CUSUM charts using auxiliary information
Source: PLoS One. 2023 Sep 15;18(9):e0290727. doi: 10.1371/journal.pone.0290727 (PMC10503744; doi:10.1371/journal.pone.0290727)
Supplement: Attachment — Submitted filename: response to the reviewers (1).docx [file pone.0290727.s004.docx]

**Reply to Reviewer’s Comments.**

**Manuscript Title:** Enhanced Performance of Mixed HWMA-CUSUM charts using Auxiliary Information

We are thankful to the reviewers and Editor(s) for their valuable comments and suggestions, which helped us improve the manuscript.

Reviewer 1 and Reviewer 2 have already given their acceptance and no questions were raised in the second review

**3^rd^ Reviewer’s Comments and Replies**

**Respected Reviewer**

Following are the comments and the possible replies from the author(s)

1. Consider Use of English language editor to improve language**:**

**Reply:** Complying with your kind suggestion, we have improved the language of the article and corrected the mistakes. Grammatical errors have been removed, equations are corrected, and errors are identified and rectified in the manuscript.

2. The motivation of this manuscript is very week. You cannot introduce a control chart because others introduced similar charts. Moreover, it is stated in the literature that the HWMA chart performs better for small shifts in the process parameter. Is there any need to combined it with the CUSUM and afterwards use an AIB to improve it again? What do you want to achieve? Did you consider the complexity of the resulting chart?

**Reply:** HWMA performs better than other charts for small shifts; combining it with the CUSUM chart improves its performance for both larger and smaller shifts. Auxiliary variable further improves the results. As in the recent age of development, improvements in quality assurance techniques are the need of the hour. In context, we have developed a new chart showing visible improvement in detecting shifts. Even a minute change and deviation in the quality can be a big hurdle in many industrial processes like lifesaving drugs, substrate manufacturing, missile equipment, etc. In some industrial and manufacturing processes, the auxiliary information is also recorded along with the information on the under-study variables for different other tasks. This information can be used to improve the control chart design without imparting any additional financial burden to the entrepreneur. We can use this information for the improvement of design. Our proposed chart is shown to have improved results compared to its counterparts and is useful for high-quality monitoring in different industrial applications. (see section 1..introduction.paragraph 2).

3. It looks like the authors did not check the formulas properly. There are many mistakes. Below Equation (1), what do y ̅_m and t? I think they were used by mistake. Check other equations and derivations as well.

**Reply:** We have made corrections to the formulae and other typing mistakes.

4. Knoth et al. (2021) advised against using the HWMA chart. They stated that the HWMA chart performs worst in steady-state and it is not recommended in practice. The same authors advised against the use of AIB chart in another article. Since both procedures are criticized, do you think that the resulting chart will be acceptable? Elaborate on this. **Reply:** Since the objection has been raised, different scholars have answered to this objection. Abbas (2022) answered the objection to using HWMA for steady-state applications. He suggested carefully choosing HWMA parameters can provide good results even for the steady state. So HWMA can be applied with a careful choice of parameter.

Also, Knoth’s objection to using auxiliary information is to use it when it is highly correlated. So as per the definition of auxiliary information, it should be correlated with the study variable; we also suggest using the proposed design only when there is a high correlation between the under-study variable and the auxiliary variable.

It is also shown that the efficiency of the design improves with the increase in the correlation coefficient value.

5. I recommend the investigation of the proposed MHCAIB chart in terms of the conditional expected delay (CED) and compare it to its counterparts considered in this manuscript in terms of the CED properties.

**Reply:** Thanks for your kind suggestion. We have started working on this idea, and it will be presented in future work.

**Thanking you in anticipation.**

**Regards**
